# Condensins are Required for Maintenance of Nuclear Architecture

**DOI:** 10.3390/cells3030865

**Published:** 2014-08-22

**Authors:** Carolyn M. George, Julianna Bozler, Huy Q. Nguyen, Giovanni Bosco

**Affiliations:** Department of Genetics, Geisel School of Medicine at Dartmouth, Hanover, NH 03755, USA; E-Mails: cgeorge@messiah.edu (C.M.G.); Julianna.E.Bozler.GR@dartmouth.edu (J.B.); Huy.Q.Nguyen.GR@dartmouth.edu (H.Q.N.)

**Keywords:** mechanical forces, condensin, lamin, nuclear architecture, chromatin

## Abstract

The 3-dimensional spatial organization of eukaryotic genomes is important for regulation of gene expression as well as DNA damage repair. It has been proposed that one basic biophysical property of all nuclei is that interphase chromatin must be kept in a condensed prestressed state in order to prevent entropic pressure of the DNA polymer from expanding and disrupting the nuclear envelope. Although many factors can contribute to specific organizational states to compact chromatin, the mechanisms through which such interphase chromatin compaction is maintained are not clearly understood. Condensin proteins are known to exert compaction forces on chromosomes in anticipation of mitosis, but it is not known whether condensins also function to maintain interphase prestressed chromatin states. Here we show that RNAi depletion of the N-CAP-H2, N-CAP-D3 and SMC2 subunits of human condensin II leads to dramatic disruption of nuclear architecture and nuclear size. This is consistent with the idea that condensin mediated chromatin compaction contributes significantly to the prestressed condensed state of the interphase nucleus, and when such compaction forces are disrupted nuclear size and shape change due to chromatin expansion.

## 1. Introduction

The nucleus of interphase cells must be able to contain the DNA polymer in a manner that safeguards the genome while also accommodating the 3-dimensional spatial organization. It is thought that one mechanism by which many DNA polymers can fit into a small nucleus is through prestressed nuclear organization, relying in part on a higher order of chromatin packaging to confer mechanical stability [1,2]. The first level of DNA organization is that of the nucleosome, in which 146 base pairs of DNA are coiled around a histone octamer [3]. Nucleosomes are bundled into 10nm chromatin fibers and subsequently organized into 3-dimensional chromatin domains [4,5,6]. Further genome compaction is achieved with the activity of global regulators of chromatin structure, resulting in a compact yet dynamic genome. It is thought that this higher order chromatin condensation is coupled with external forces from the nuclear lamina and cytoskeleton, and collectively these forces maintain the nuclear architecture and resist the entropic forces of the DNA polymer [1,2].

A complete picture of how higher order chromatin compaction is maintained, and how forces that drive remodeling of chromatin are coordinated with forces that maintain nuclear structure remains elusive. Histone dependent compaction has been identified as one component of this mechanism [7]. It is likely that additional proteins are participating in this nuclear stabilization. For instance, condensins are known to exert compaction forces on chromatin in preparation for mitosis, with condensin II activity persisting throughout interphase. Condensin II complexes contain SMC2/SMC4 ATPases and chromosome associating proteins (CAP) CAP-H2, CAP-D3, and CAP-G2 [8]. Hyperactivation of condensin II can drive axial compaction in interphase [9,10,11]. In Drosophila, this interphase hypercondensation can lead to forces that drive extensive nuclear envelope remodeling [12], reminiscent of nuclear defects found in human cells with a variety of lamin mutations [13]. Conversely, when condensin II specific subunits are depleted, interphase chromatin length increases [10,11,14,15]. Regulation of condensin II therefore may be a means of modulating global compaction for the purposes of conferring mechanical stability to the nucleus [12,16]. An extreme case of condensin II mediated interphase compaction and changes in nuclear size is observed in quiescent mouse T-cells that decondense chromatin as they are activated [17].

The model of the prestressed nucleus proposes that stability of chromatin condensation state is essential for maintenance of the nuclear architecture since enzymatic decondensation of chromatin leads to entropic swelling of isolated nuclei [1]. However, the role of biologically regulated condensation in intact cells is less clear. In the context of a cell, the destabilization of the nucleus may result in unequal expansion and manifest in aberrant nuclear architecture as is seen in syndromes such as Emery-Dreifuss muscular dystrophy, Hutchinson-Gilford Progeria Syndrome and a number of different cancers that are primarily characterized by nuclear defects [13,18,19].

Given the importance of nuclear architecture for gene regulation and cellular function, it is critical to understand the factors contributing to its stability. Here we explore the role of condensin II in the mechanical stability of nuclei in cultured Drosophila and human cancer cell lines. We show that destabilization of the nucleus through knock-down of a biologically relevant regulator of chromatin condensation in cells, condensin II, results in nuclear swelling and nuclear morphology defects. These data support a model where condensin II mediated chromatin compaction contributes to a prestressed condensed state of the interphase nucleus.

## 2. Experimental Section

### 2.1. Drosophila Cells and RNAi

Drosophila Kc167 and S2 cell culture, *in vitro* dsRNA synthesis, and RNAi treatments were performed as previously described [20]. RNAi was performed with 10 µg of dsRNA on 50%–90% confluent cells, with fresh 1ml of media and 10 µg dsRNA every other day for 6 days. Control (SK) dsRNA was made by amplifying a sequence of the pEGFP-N1 vector (Takara Bio Inc., Mountain View, CA, USA) using primers 5'-*TAATACGACTCACTATAGGG*CGCTTTTCTGGATTCATCGAC-3' and 5'-*TAATACGACTCACTATAGGG*TGAGTAACCTGAGGCTATGG-3'; PCR products for Cap-H2 dsRNA was made from genomic DNA template using primers 5'-*TAATACGACTCACTATAGGG*ACCGGAGAAAAACGAGCGCAGGCC-3' and 5'-*TAATACGACTCACTATAGGG*GGGATCCACTCTCGTGC-3'. Italic sequences denotes T7 polymerase promoter. PCR products were used with T7 RiboMAX Express Large Scale RNA Production System kit (Promega, P1320). dsRNA concentration was calculated using agarose gels and densitometry (NIH ImageJ).

### 2.2. Immunofluorescence of Drosophila Cells

Cells were immunostained as previously described [20]. Cells were plated onto Concanavalin-A (Sigma-Aldrich, St. Louis, MO, USA) coated cover slips, adhered for 20 min, fixed with 10% formaldehyde (Ted Pella, Redding, CA, USA) in PBS for 5 min, washed with PBS, then 0.1% PBS/Triton to permeabilize. Cells were blocked (5% normal goat serum (Sigma-Aldrich), 0.1% PBS/Triton, and 1 mM Sodium Azide (Sigma-Aldrich) for 1 h. Primary antibodies were diluted in block solution and coverslips incubated for 1 h at room temperature. Mouse anti-Lamin (Dm_0_) ADL 84.12 was at 1:200 (Developmental Hybridoma Bank, Iowa City, IA, USA). Cells were washed with PBT (0.1% Triton PBS, Sigma, St. Louis, MO, USA) 3× 5 min. Secondary antibody (Cy-3, Jackson ImmunoResearch Laboratories, West Grove, PA, USA) diluted in block solution (1:500) and coverslips were incubated with secondary for 2 h, washed with PBT 3× 5 min, then with PBS 5 min, all at room temperature. 4',6-Diamidino-2-Phenylindole, Dihydrochloride (DAPI) (Life Technologies, Grand Island, NY, USA) was added (1 µg/mL in PBS) for 10 min, then washed 2× in PBS for 5 min. Coverslips were mounted on slides with Vectashield (Vector Labs, Burlingame, CA, USA), sealed with nail polish, and stored at −20 °C. Images were obtained on a Nikon A1RSi confocal microscope with Plan Apo 100× 1.49 NA oil objective and Nikon Elements 4.0 software and processed using ImageJ (NIH).

**Figure 1:**
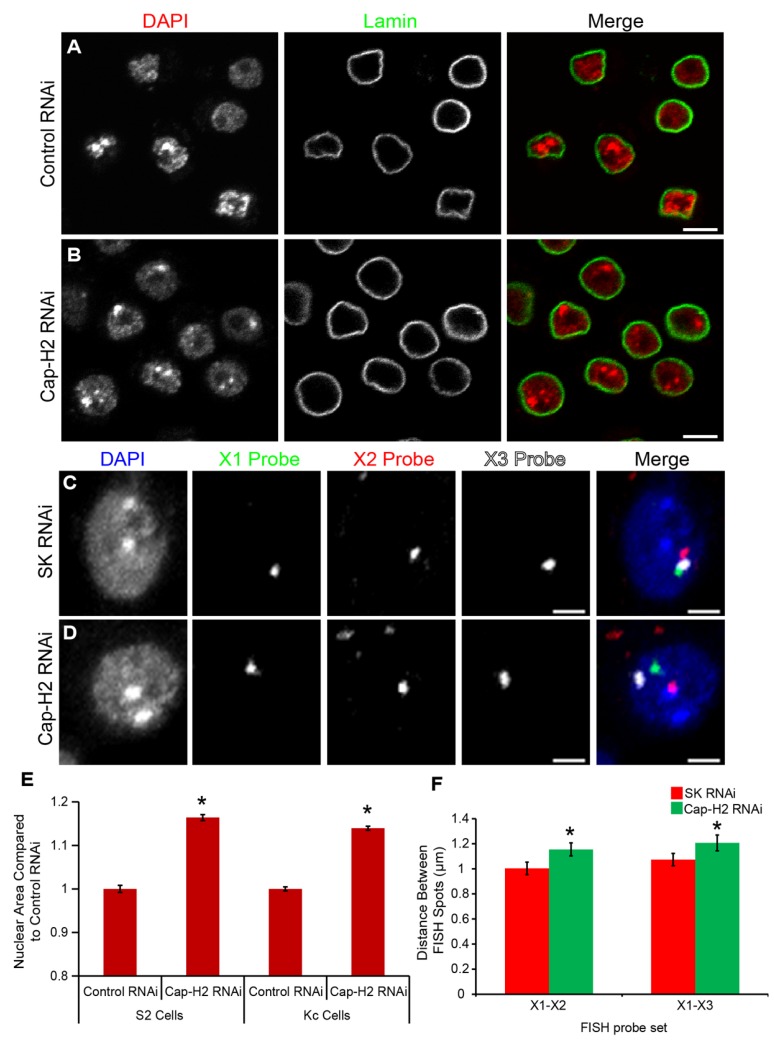
Drosophila condensin II subunit Cap-H2 maintains nuclear size. (**A**,**B**) Control RNAi (**A**) or Cap-H2 RNAi (**B**) treated *Drosophila* cultured Kc cells counterstained with DAPI to visualize DNA (red) and immunostained for Lamin (green). Scale bar, 5 µm; (**C**,**D**) Control (SK) RNAi or Cap-H2 RNAi treated cells and labeled with three different X-chromosome FISH probes. Scale bar, 2.5 µm; (**E**) Graph showing average nuclear area (DAPI signal) of RNAi treated *Drosophila* cultured cells (S2 and Kc cells). Cap-H2 RNAi significantly increases nuclear area in both S2 and Kc cells. * *p <* 1.42 × 10^−52^; (*n =* 3300–9000 cells; two-tailed T-test assuming unequal variance). Error bars indicate SEM; (**F**) Distances in microns (μm) between two FISH probe pairs (X1-X2 and X1-X3) measured in 3-dimensional space in Kc167 cells treated with control (SK) or Cap-H2 RNAi. * *p <* 0.05; (*n =* 50 cells for each condition; two-tailed T-test assuming unequal variance). Error bars indicate SEM.

Nuclear area was measured with “IdentifyPrimaryObjects” then “MeasureObjectSizeShape” function in CellProfiler Cell Image Analysis Software (Broad Institute, Cambridge, MA, USA)[21,22]. Micrographs were captured on a Nikon E800 Epifluorescence microscope, Plan Fluor 20× 0.5 NA objective with Olympus DP software. Single z-slices of DAPI stained Drosophila cells were used for nuclear area analysis measured in pixels. Cells from the same experiment were imaged using identical settings (exposure). Thresholding was performed equally for all images. Graphs in Figure 1 represent area measurements from 2 biological replicates and each RNAi treatment normalized to pixel area from control RNAi treated cells. Per cent change in nuclear size was calculated as follows: [(pixel area of Cap-H2 RNAi nuclei)-(pixel area of control RNAi nuclei)]/(pixel area of control RNAi nuclei) × 100.

### 2.3. Fluorescent In-Situ Hybridization (FISH)

Cultured cell FISH was performed as previously described [12]. Cells were plated onto Con-A coated coverslips in a well of a 6-well tissue culture plate for 20 min and allowed to adhere to coverslips at room temperature. Coverslips were then washed with 1× PBS and fixed in 10% Formaldehyde/PBS for 10 min at RT. Coverslips were then washed once with PBS and permeabalized in 0.1% PBS/Triton for 10 min. Cells were then washed in CSK buffer (10 mM HEPES, 100 mM NaCl, 3 mM MgCl_2_, 300 mM Sucrose, and Phenylmethanesufonyl fluoride (PMSF)) for 10 min and Ribonuclease A (100 ug/mL) for 1 h at RT. Cells were then washed with 0.1N HCl for 5 min and taken through ethanol series for 5 min each (70%, 90%, then 100%). One 2× SSCT wash was performed and cells were pre-hybed in 50% formamide/2× SSCT at 37 degrees for 2 h. FISH probe (1–2 µL each probe) was then added to hybridization solution (total 25 µL) and this mixture was denatured at 95 °C for 2 min and snap cooled in an ice bath. Probe mixture was then added onto microscope slide and coverslips were inverted onto the microscope slide. Coverslips were then sealed with rubber cement and slide/coverslip was denatured at 93 °C on a heat block for 3 min. Slides were then placed in humid chamber and hybridized overnight at 37 °C. After hybridization was complete, coverslips were detached from slides by immersing in 50% formamide/2× SSCT with shaking for 10 min. Coverslips were then placed into 6-well tissue culture plates and washed three times for 30 min at 42 °C. Ten minute washes at 42 °C were then performed with 40% then 20% formamide in 2× SSCT. Three 2× SSCT washes were performed for 5 min each at RT on shaker. Cells were then counterstained with DAPI (1 µg/mL) in PBS for 10 min at RT. Coverslips were then washed two times for 10 min at RT in PBS. Cells were then mounted with Vectashield and sealed with nail polish.

### 2.4. FISH Probe Preparation

Euchromatic FISH probes were made as previously described [11,12] from BAC clones (Children’s Hospital Oakland Research Institute BACPAC Resources) as follows: X1, BACR30C13 and BACR18F10; X2, BACR20K01 and BACR35A18; X3, BACR11C13. BAC clones were mapped and picked by using the UCSC genome browser [23]. BAC clones were cultured and DNA was purified using QIAGEN Plasmid Midi Kit (Qiagen 12143, Hilden, Germany). Purified BAC DNA was amplified using Whole genome amplification kit (Sigma-Aldrich WGA1), 20 µg of amplified DNA was then digested using a cocktail of AluI, Rsa, MseI, MspI, HaeIII, and BfuCl (New England BioLabs, Ipswich, MA, USA) overnight at 37 °C, ethanol precipitated, and resuspended in 36 µL ddH_2_O. DNA was denatured at 100 °C for 1 min, and then 3'-end-labeled with unmodified aminoallyl dUTP and terminal deoxynucleotidyl transferase (Roche, Mannheim, Germany). After incubating for 2 h at 37 °C, 5 mM EDTA was added to terminate the reaction. DNA was ethanol precipitated, resuspended in 10 μL ddH2O, and then conjugated to fluorophores using ARES Alexa Fluor DNA labeling kits (A-21665, A21667, and A-21676; Life Technologies) for 2 h, according to the manufacturer’s instructions. Probes were then cleaned up using Qiagen PCR clean up kit (Qiagen, Hilden, Germany), ethanol precipitated, and resuspended in 10 µL EB buffer (Qiagen).

### 2.5. Microscopy/Image Analysis

FISH images were obtained on a Nikon A1RSi confocal microscope using either a Plan Apo 60× 1.4 NA or a Plan Apo 100× 1.49 NA oil immersion objective using the Nikon Elements 4.0 software package. Micrographs were processed using Nikon Elements or ImageJ (NIH). FISH spot counts were performed on maximum z-projections from z-stack images using the counting software in Nikon Elements. 3D FISH distance measurements were performed manually in Nikon Elements using the 3D distance measuring tool by scanning through each Z-slice. The centroid of each FISH signal would be marked and the shortest 3D pairwise distance would then be calculated.

### 2.6. Human Cell Culture

HeLa, HCT116 and U2-OS cells were generously provided by the Compton laboratory at Dartmouth College and maintained at 37 °C, 5% carbon dioxide in Dulbecco’s Modified Eagle’s Medium, 10% fetal bovine serum and 1% penicillin-streptomycin (Corning 10-013, 35-015-CV, 30-001-CI, Tewksbury, MA, USA). Cells were in 100 mm culture dishes and passaged at a dilution of 1:10 to 1:20 when confluency reached 70%–90% using 0.05% Trypsin/0.53 mM EDTA in HBSS (Corning 25-051-CI).

### 2.7. siRNA of Condensin II in Human Cells

HeLa, HCT116 or U2-OS cultures were harvested at 70%–80% confluence and 1/20 were used to transfect with siRNA (300 pmol for HeLa; 150 pmol for HCT116 and U2-OS) to NCAPH2, NCAPD3, or SMC2, and a scrambled siRNA (ThermoScientific, SMARTpool siGENOME, M-016186-01-0005, M-026539-01-0005, M-006836-01-0005, D-001206-14-05), each consisting of four siRNA. siRNA was nucleofected (Nucleofector 2b, Lonza, program I-013) with nucleofection kit V (VACA-1003, Lonza/Amaxa, Walkersville, MD, USA). Cells were divided 1:2 and plated into two wells of a 6-well dish and harvested after two days. Contents of one well were used for immunoblot and the other contained a 15 × 15 mm coverslip for immunofluorescence. Nucleofection efficiency in HeLa cells was close to 100%, determined by co-transfection with pmax-GFP. Knockdown was confirmed in each experiment by immunoblot. HCT116 and U2OS (not HeLa) cells were co-transfected with 1 μg of pEGFP-D50 Lamin A (17653, Addgene, Cambridge, MA, USA) with siRNA. Efficiency was assessed by GFP signal.

### 2.8. Immunoblot

Following two days incubation, transfected cells were harvested by chilling on ice and rinsing each well 3× with PBS. 200 µL of 2× sample buffer (1 M Tris pH 6.8, 50% glycerol, 10% SDS, 0.5% bromophenol blue, 10% β-mercaptoethanol) was added and cells were removed from the well surface and suspended using a scraper. Cell suspensions were transferred to a 1.5 mL tube and were lysed by sonication (2 × 15 s pulses at 30% amplitude, Branson SLPe sonicator with 2 min on ice between pulses). Lysates were used immediately for immunoblot or were divided into 50 µL aliquots, frozen in liquid nitrogen, and stored at −80 °C. Lysates used for immunoblot were boiled for 10 min, cooled 2 min on ice prior to loading of 15 µL each sample on an 8% SDS-PAGE mini gel. Gels were run at 100 V for 1 h, then transferred (Bio-Rad mini transfer) to nitrocellulose membrane (GE Healthcare, Piscataway, NJ, USA, 10600002) on ice at 100 V for 1 h. Transfer was confirmed by Ponceau stain. Blots were rinsed with 1× TBS-T, overnight at 4 °C in 10% Carnation instant milk/TBS-T, then incubated with 2% milk/TBS-T containing primary antibody at room temperature for 1–2 h. Primary antibodies were 1:1000 of rabbit anti-NCAPH2 (AFR205-4L, a gift from T. Hirano, RIKEN Institute [24]), 1:1000 of rabbit anti-NCAPD3 (Abgent AP16786B, San Diego, CA, USA), or 1:1000 of mouse anti-α-Tubulin (Sigma, T9026). Blots were washed 3× 10 min in 1× TBS-T and probed with secondary antibody (1:5000 HRP-anti-rabbit or anti-mouse, Jackson ImmunoResearch, West Grove, PA, USA) in 2% milk/TBS-T, washed 3× in 1× TBS-T, treated with Pierce ECL Blotting Substrate or Bio-Rad Clarity ECL for 5 min, exposed to Ewen-Parker Blue (EBA45) film, and processed on a XO-MAT. Scanned films were quantified using Image J software. NCAPH2 or NCAPD3 signals were normalized to Tubulin signal. Three or more biological replicates were averaged and error bars represent SEM.

### 2.9. Immunofluorescence and Nuclear Abnormality Analysis

Following two days incubation, transfected cells on coverslips were rinsed 3x in PBS and fixed in 3.7% formaldehyde/PBS for 15 min at room temperature, followed by 3× 5 min washes in PBS and either storage at 4 °C or immediate overnight incubation (2.5% BSA/0.3% Triton X-100/PBS). Fixed cells were probed with 1:200 goat anti-Lamin A/C (Santa Cruz N-18, SC-6215), 1% BSA/0.3% Triton X-100/PBS for 1–2 h at room temperature, washed 3× in 1× PBS, then probed with either 1:200 donkey anti-goat-Cy2 or anti-goat-Cy3 (705-225-147, 705-165-147, Jackson ImmunoResearch, West Grove, PA, USA) for 1–2 h. After 3× washes in PBS cells were stained with 1 µg/mL DAPI in PBS for 5 min, washed 3× for 5 min in PBS. Coverslips were mounted to slides on VectaShield (Vector Labs, Burlingame, CA, USA). Nuclear shape in HCT116 and U2OS cells was visualized by co-transfecting siRNA with 1 μg of pEGFP-D50 Lamin A (17653, Addgene, Cambridge, MA, USA). Nuclear shape was scored by visual inspection using a Nikon E800 microscope. To eliminate bias, samples were scored blindly by hiding the identities of samples until all slides were scored. Nuclear shape was rated by placing 250 nuclei per sample into four categories. Normal: round/oval shaped, smooth edges. Mild: round/oval or bean-shaped with ruffled edges. Moderate: not round/oval, has numerous ruffles and/or one to two folds, dents, or holes. Severe: numerous folds, deep dents, large holes, or is multi-lobed. For each sample the fraction of total nuclei falling into each category was plotted with SEM. A chi-square goodness of fit test in R (3.0.2 “Frisbee Sailing”) was used to compare each sample distribution to control siRNA-treated cells to determine significance for all three human cell lines. The per cent change in nuclear size was calculated exactly as described above for Drosophila cells.

## 3. Results and Discussion

### 3.1. The Prestressed Nucleus and Compaction Forces

In vertebrate cells condensin II activity has been shown to induce axial shortening of chromosomes as they prepare to enter mitosis [25,26]. Recently, Manning and Dyson have shown that depletion of human NCAP-D3 leads to a loss of interphase compaction [15], while Fazzio and Panning have shown that RNAi depletion of condensins in mouse embryonic stem (ES) cells leads to enlarged interphase nuclei and loss of chromatin compaction [14]. Maintenance of hypercondensed chromatin in mouse quiescent T-cells also requires condensin II Cap-H2 activity [17]. Drosophila condensin II is also required for maintenance of interphase chromosome compaction [10,11]. By contrast, hyper-activation of condensin II in interphase leads to remodeling of the nuclear envelope, and it has been proposed that condensin mediated mechanical forces act on envelope-tethered chromatin to pull envelope structures toward the interior of the nucleus [12,16]. These observations suggest that condensin II mediated forces have a significant role in interphase nuclear architecture and genome compaction. It is therefore possible that compaction forces contribute to the mechanical stability of the nucleus in interphase.

Interphase chromatin is thought to be in a prestressed state due to condensation [27], and because the nucleus is surrounded by cytoplasm, the nuclear envelope is subjected to mechanical forces both outside and inside the nucleus. This is in part due to tensile forces of the actin cytoskeleton that use physical linkages on the outer envelope. These forces are countered by chromatin condensation, acting through chromatin-envelope tethers and chromatin-matrix attachments [1,28], each force pulling in opposite direction as they converge on the nuclear envelope [2]. These forces are paired with the compressive action of microtubules and entropic force of the DNA polymer itself, together creating a balanced web of counteracting forces upon the nuclear envelope. Mechanical force applied to the nucleus can have direct molecular consequences, thus linking mechanotransduction to functional roles of specific processes within the nucleus [29]. Disruption of one or more of these counterbalancing forces may lead to defects in nuclear shape and function, such as is observed in laminopathies and other related human diseases, and consequent misregulation of gene expression [30].

### 3.2. Condensins Regulate Nuclear Size in Drosophila Cultured Cells

That chromatin normally exists as a compressed spring-like polymer has been tested by a variety of chemical and enzymatic treatments of isolated nuclei, demonstrating that chromatin decondensation can lead to increased nuclear size and nuclear rupture [1]. However, it is not known to what extent interphase condensin activity contributes to this process. Because condensin has been shown to maintain interphase chromosome compaction in Drosophila, mouse and human cells [10,11,12,14,17,25,26], we wished to test whether condensin II contributes to the prestressed state of the nucleus. If condensin II compaction forces contribute to spring-like compression of chromatin, then RNAi depletion of condensin II activity predicts that nuclear size and/or shape should change. We used Drosophila cultured Kc167 and S2 cells treated with RNAi to Cap-H2, the rate limiting subunit of condensin II [9,10]. Cap-H2 depleted nuclei were found to increase in size by 14% ± 0.5% in Kc cells and 16% ± 0.7% in S2 cells, relative to control RNAi treated cells (Figure 1). This was found to be a significant increase in nuclear size. However, under close inspection we were not able to detect any observable defects in nuclear shape in either Kc or S2 cultured cells that were depleted of Cap-H2 (Figure 1A,B,E). Using two sets of FISH probes on the X-chromosomes we measured the change in distance between two regions approximately 2Mb apart. For both regions on the X-chromosomes cells RNAi depleted of Cap-H2 displayed a significant increase in interphase chromatin axial length (Figure 1C,D,F). Thus, changes in nuclear size are accompanied by axial expansion of chromatin after Cap-H2 depletion.

### 3.3. Condensins Regulate Nuclear Size in HeLa Cells

**Figure 2:**
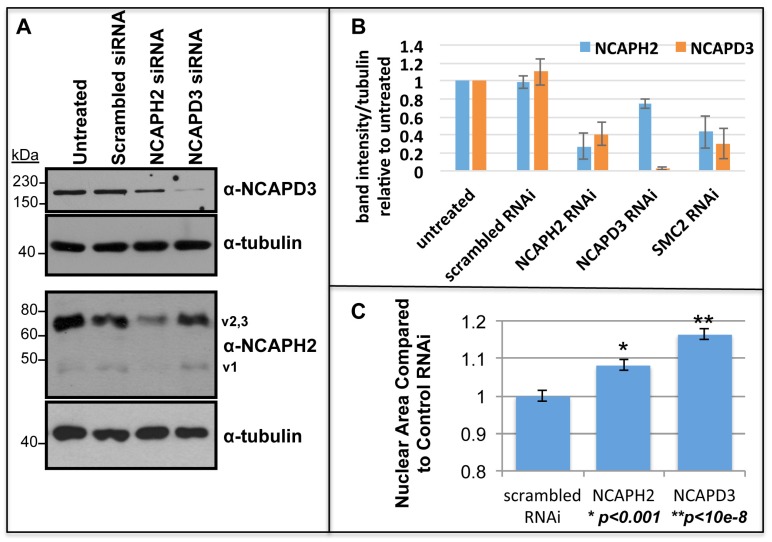
siRNA effectively depletes human condensin II subunits. (**A**) Representative immunoblot of HeLa extracts from cells treated with indicated siRNA. Top panel show endogenous NCAPD3. Cells in bottom panels were co-transfected with splice variant 1 of NCAPH2 and show knockdown of this variant and endogenous variant 2 or 3, which cannot be distinguished in this experiment; (**B**) Quantification of NCAPH2 and NCAPD3 knockdown by immunoblot normalized to tubulin intensity. Average intensities for RNAi treated samples are plotted relative to untreated samples; (**C**) Condensin depletion results in increased nuclear size. HeLa cells treated with scrambled siRNA or siRNA targeting NCAPH2 or NCAPD3 and average nuclear area is shown, normalized to scrambled siRNA. For each 231 nuclei were measured, * *p <* 0.001 and ** *p <* 10^−8^, two-tailed T-test assuming unequal variance. Error bars represent S.E.M.

To test how condensin II function affects nuclear size in human cells, we used siRNA to different condensin subunits. Using siRNA targeting either NCAP-H2 or NCAP-D3 transfected into HeLa cells we were able to achieve significant depletion of each of these proteins (Figure 2A,B). For NCAP-H2, we observed that siRNA was able to reduce three different NCAP-H2 protein isoform variants (Figure 2A). Interestingly, siRNA targeting one subunit also had the effect of depleting protein levels of the other, and this effect was most dramatic in siRNA targeted to NCAP-H2 that led to a decrease in NCAP-D3 protein. To further validate this effect, we used siRNA to a third subunit, SMC2, and also found a decrease in both NCAP-H2 and NCAP-D3 proteins (Figure 2B).

Untreated cells and control scrambled siRNA treated cells repeatedly failed to produce any detectable decrease in either NCAP-H2 or NCAP-D3 (Figure 2). This is consistent with the idea that stability of the individual condensin subunits necessitates formation of the multi-subunit complex, and stoichiometric changes in one subunit may cause instability of other subunits that cannot form complexes. A previous report showed this same effect, as RNAi depletion of Smc2 led to a significant decrease in NCAP-D3 protein [31].

To quantify changes in HeLa cell nuclear size, siRNA transfected cells were fixed and stained for DNA and nuclear lamin-A/C. Compared to cells treated with control scrambled siRNA, those treated with siRNA to NCAP-H2 increased in size by 8% ± 1.5%, and cells treated with siRNA to NCAP-D3 increased in size by 16% ± 1.4% (Figure 2C). Both these changes are significant. It is unclear why the change in size after siRNA to NCAP-H2 is so small, but one possible explanation is that the NCAP-H2 protein depletion was not very efficient (Figure 2A,B).

### 3.4. Condensins Regulate Nuclear Shape in Human Cells

**Figure 3:**
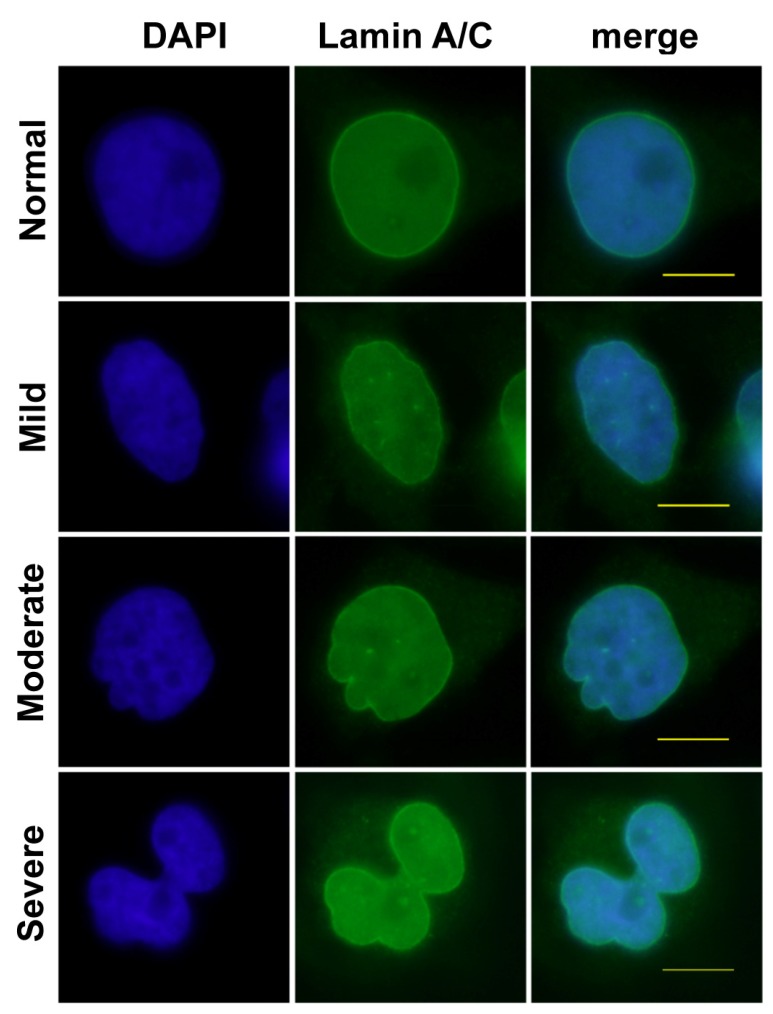
Four categories of nuclear morphology. Nuclear shape of RNAi treated HeLa cells as seen by Lamins A/C (green), DNA (blue) and placed into four categories ranging from “Normal” round/oval nuclei; nuclei having few or “Mild” abnormal features; nuclei with obvious or “Moderate” amount of abnormal features; “Severe” with numerous abnormal features and overall non-round/oval shape. See methods for details. Scale bar, 5 μm.

**Figure 4:**
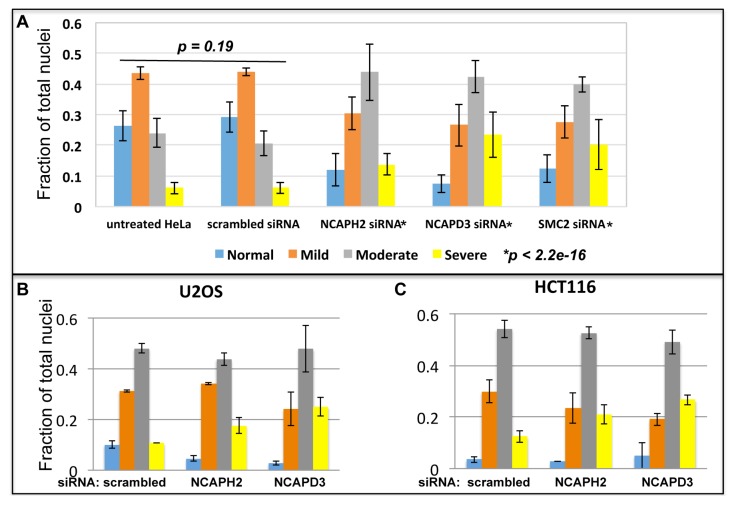
Condensin II is required for nuclear shape maintenance. (**A**) HeLa cells untreated, treated with scrambled siRNA or siRNA targeting NCAPH2, NCAPD3 or SMC2. Nuclei were scored blind and placed into four categories (as in Figure 3). Each represents the average of three or more independent experiments and error bars represent SEM. Distributions of nuclear shape for each treatment were compared to negative control siRNA (scrambled) using a chi-square goodness of fit test and p values are shown; (**B**,**C**) U20S and HCT116 cells treated with scrambled siRNA or siRNA targeting NCAPH2 and NCAPD3. In this experiment cells were also co-transfected with Lamin-GFP.

Changes in morphology were quantitated by placing nuclei into four different categories (normal, mild, moderate and severe, Figure 3). To control for nuclear defects caused by the transfection and/or introduction of siRNA into cells we compared nuclei in untreated HeLa cells to those that were transfected with scrambled siRNA molecules. Population distributions were analyzed using a chi-square goodness of fit test. We found no statistical difference between untreated and scrambled siRNA treated cell populations (*p =* 0.1923, Figure 4A). However, cells treated with siRNA targeting NCAP-H2, NCAP-D3 and SMC2 each had a significant (*p < 2.2e^−16^) decrease in the fraction of normal and mild nuclei and an increase in both moderate and severe nuclear morphologies (Figure 4A). We also observed this same trend in U2OS and HCT116 cell lines, where the fraction of nuclei with moderate and severe nuclear morphology was increased after siRNA depletion of NCAP-H2 and NCAP-D3 (Figure 4C,D). We conclude that condensin II activity is required for maintaining normal nuclear shape in HeLa, U2OS and HCT116 human cell lines.

It is interesting to note the difference in nuclear shape after condensin II depletion in Drosophila cells compared to human cells. In two different *Drosophila* cultured cell lines, nuclear size increased after condensin II depletion, but there was no observable abnormality in nuclear shape (Figure 1). One possible interpretation of these data is that *Drosophila* condensins are not critical for maintenance of nuclear shape. Alternatively, it is possible that other tissue types and/or developmental contexts to require condensin mediated compaction forces to regulate nuclear shape. Indeed, even small increases in *Drosophila* condensin II activity *in vivo* and in cultured cells leads to dramatic changes in nuclear shape [12]. In contrast to *Drosophila* cells, depletion of condensin II in three different human cell lines led to changes in nuclear shape (Figure 3 and Figure 4).

This study only examines human cancer cell lines, and therefore it is possible that the changes in nuclear shape we observe after condensin II depletion is a unique property of transformed cells. For example, one or more mutations common to HeLa, HCT116 and U2OS cells could contribute to perturbations of nuclear structure after condensin II depletion. Future studies using a variety of primary human cells will be necessary to determine how general the condensin II nuclear defects are among different human cell types. We emphasize, however, that recent studies in human cells (including HeLa), mouse ES cells and Drosophila cells (Kc167) have uncovered a conserved role for chromatin architectural proteins that define chromatin domains, and this includes the condensin II protein Cap-H2 [32,33]. Thus, Drosophila and human cells (including cancer cells) seem to have similar 3-dimensional architectural features that are likely defined by a conserved chromatin protein landscape, nicely correlated with the Cap-H2 condensin II binding sites. Importantly, the increase in nuclear size we report in Figure 1 for Drosophila cells and in Figure 2 for human cancer cells is consistent with previously published data showing an increase in nuclear size of mouse ES cells after condensin RNAi depletion, and this was accompanied by measurable loss of interphase chromatin compaction [14]. Increase in nuclear size, changes in nuclear shape and decrease in chromatin compaction are also seen in mouse T-cells as they exit quiescence [17]. These observations in mouse ES and T-cells suggest that the effects of condensins on nuclear size and shape may be a general cellular function, not limited to transformed cancer cells. Nevertheless, if the nuclear size and shape changes upon condensin depletion were unique to transformed human cells, we suggest that loss of condensin function (or overexpression) in this context may still be biologically relevant since mutations of human condensin genes are known to occur in gastric cancers [34] and pyothorax-associated lymphoma [35]. Similarly, SMC2 expression can be a driver of proliferation in MYCN-amplified neuroblastoma cells [36], and SMC2 has been identified as a critical pro-mitogenic transcriptional target of the WNT signaling pathway in intestinal tumor cells [37]. These observations strongly suggest that even if the nuclear shape defects reported here are unique to transformed cancer cells, then such condensin-mediated nuclear defects may have functional consequences on those cellular processes that promote proliferation and malignancy, and some have speculated that condensins could serve as attractive targets for anti-cancer therapies [36,37,38].

### 3.5. Model of Mechanical Forces Modulating Nuclear Shape

**Figure 5:**
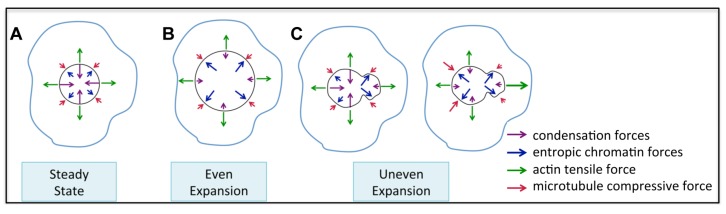
Speculative model of how condensin II depletion leads to changes in nuclear shape. (**A**–**C**) Within a cell (blue line) the nuclear envelope (black circle) has various forces (arrows) acting on it; chromatin compaction (purple), entropic chromatin forces (blue), actin tensile force (green), and compressive force from microtubules (red). In the prestress state, the forces on the nuclear envelope are balanced, creating a steady state stable nuclear architecture (**A**); Even expansion of the nucleus occurs when the prestress state is compromised through loss of chromatin compaction, while maintaining an even distribution of forces across the envelope (**B**); Alternatively, uneven expansion of the nucleus can occur when there is an asymmetrical distribution of forces (**C**). This may occur through localized loss of nuclear compaction, regional differences in the nuclear lamina, asymmetric chromatin-nuclear matrix anchors that restrict expansion, or through uneven cytoskeletal attachments and forces.

It is possible to reconcile the differences in nuclear shape we observe in human and *Drosophila* cells if the distribution of nuclear envelope structural components is homogenous in cultured Drosophila cells. In this case, decondensation would lead to expansion of the nucleus in an isometric fashion, resulting in a larger nuclear size as the nucleus expands equally in all directions. However, if structural components, such as lamins, and cytoskeletal forces are not evenly distributed along the nuclear envelope, then the expansion force of chromatin could be resisted at some regions of the envelope while other regions would be more susceptible (Figure 5). Alternatively, chromatin compaction in human interphase cells may not be equally distributed within the nucleus, and depletion of condensins may result in decondensation forces that are focused on one area of the nucleus, resulting in uneven expansion of the nuclear envelope due to asymmetrical application of internal nuclear forces. An asymmetrical distribution of chromatin-nuclear matrix anchor points within the nucleus could also contribute to uneven expansion forces, since chromatin fibers tethered to matrix attachment sites could restrict how the DNA polymer expands [1,28]. Abnormal nuclear matrix has been reported in human cancer cells [39,40], thus such aberrant matrix organization could also be contributing to condensin-mediated changes in nuclear size and shape in HeLa, HCT116 and U2OS cells analyzed here.

These are not mutually exclusive possibilities, but regardless of the cause, changes of mechanical forces on either side of the nuclear envelope without a corresponding counterbalancing force may facilitate distortions of the nuclear envelope (Figure 5). Such distortions are not necessarily detrimental, and in fact, they could be dynamic and regulated structures of functional import. For example, membrane structures, called nucleoplasmic reticulum (NR), emanating from the nuclear envelope into the interior of the nucleus, have been observed in many cell types of multiple species. These NR structures are postulated to provide greater envelope surface area that reaches the interior of the nucleus, thus facilitating nuclear import/export efficiency [41]. Additionally, changes in the curvature of lipid membranes can alter the dynamics of protein-protein interactions and stimulate signaling pathways [42]. Thus, it is interesting to speculate that condensin mediated mechanical forces inside the nucleus drive changes in nuclear shape, including curvature of envelope membrane, and may be a highly regulated process that is important for cellular function. It is also possible that changes in gene expression due to condensin II depletion could have an indirect effect on nuclear structure.

## 4. Conclusions

We show that depletion of condensin II subunits in Drosophila Kc and S2 cells and human HeLa cells leads to increased nuclear size. In three different human cancer cell lines depletion of condensin II leads to dramatic changes in nuclear shape. These observations are consistent with the idea that mechanical compaction of chromatin, in conjunction with other forces, contributes to the maintenance of nuclear size and shape. Mechanistically, it is still not known how interphase chromatin is compacted so as to decrease its axial length and lateral width. The observations reported here suggest that chromatin–chromatin interactions, chromatin-envelope and chromatin-nuclear matrix interactions may be at play while mechanical forces inside the nucleus and cytoskeletal forces outside the nucleus cooperate in regulating nuclear shape and function.
